# Transmission Dynamics and Prospects for the Elimination of Canine Rabies

**DOI:** 10.1371/journal.pbio.1000053

**Published:** 2009-03-10

**Authors:** Katie Hampson, Jonathan Dushoff, Sarah Cleaveland, Daniel T Haydon, Magai Kaare, Craig Packer, Andy Dobson

**Affiliations:** 1 Department of Ecology and Evolutionary Biology, Princeton University, Princeton, New Jersey, United States of America; 2 Department of Animal and Plant Sciences, University of Sheffield, Western Bank, Sheffield, United Kingdom; 3 Department of Biology, McMaster University, Hamilton, Ontario, Canada; 4 The Roslin Institute/ Royal (Dick) School of Veterinary Studies, University of Edinburgh, Easter Bush, Roslin, Midlothian, United Kingdom; 5 Boyd Orr Centre for Population and Ecosystem Health, University of Glasgow, Glasgow, United Kingdom; 6 Centre for Infectious Diseases, School of Biological Sciences, University of Edinburgh, Edinburgh, Midlothian, United Kingdom; 7 Department of Ecology, Evolution and Behavior, University of Minnesota, St. Paul, Minnesota, United States of America; Centers for Disease Control and Prevention, United States of America

## Abstract

Rabies has been eliminated from domestic dog populations in Western Europe and North America, but continues to kill many thousands of people throughout Africa and Asia every year. A quantitative understanding of transmission dynamics in domestic dog populations provides critical information to assess whether global elimination of canine rabies is possible. We report extensive observations of individual rabid animals in Tanzania and generate a uniquely detailed analysis of transmission biology, which explains important epidemiological features, including the level of variation in epidemic trajectories. We found that the basic reproductive number for rabies, R_0_, is very low in our study area in rural Africa (∼1.2) and throughout its historic global range (<2). This finding provides strong support for the feasibility of controlling endemic canine rabies by vaccination, even near wildlife areas with large wild carnivore populations. However, we show that rapid turnover of domestic dog populations has been a major obstacle to successful control in developing countries, thus regular pulse vaccinations will be required to maintain population-level immunity between campaigns. Nonetheless our analyses suggest that with sustained, international commitment, global elimination of rabies from domestic dog populations, the most dangerous vector to humans, is a realistic goal.

## Author Summary

Canine rabies has been successfully eliminated from Western Europe and North America, but in the developing world, someone dies every ten minutes from this horrific disease, which is primarily spread by domestic dogs. A quantitative understanding of rabies transmission dynamics in domestic dog populations is crucial to determining whether global elimination can be achieved. The unique pathology of rabies allowed us to trace case-to-case transmission directly, during a rabies outbreak in northern Tanzania. From these unusual data, we generated a detailed analysis of rabies transmission biology and found evidence for surprisingly low levels of transmission. We also analysed outbreak data from around the world and found that the transmission of canine rabies has been inherently low throughout its global historic range, explaining the success of control efforts in developed countries. However, we show that when birth and death rates in domestic dog populations are high, such as in our study populations in Tanzania, it is more difficult to maintain population-level immunity in between vaccination campaigns. Nonetheless, we conclude that, although the level of vaccination coverage required is higher than would be predicted from naïve transmission models, global elimination of canine rabies can be achieved through appropriately designed, sustained domestic dog vaccination campaigns.

## Abstract 3

Low transmission rates in rural Africa and elsewhere suggest that sustained, appropriately designed domestic dog vaccination campaigns could lead to global elimination of canine rabies, which would save over 50,000 human lives per year.

## Introduction

Rabies has been one of the most feared diseases throughout human history and has the highest human case-fatality proportion of any infectious disease [1,2]. Every year over 7 million people receive post-exposure prophylaxis, and an estimated 55,000 people die from rabies [3] (more than yellow fever, dengue fever, or Japanese encephalitis [4]). Over 99% of these deaths occur in developing countries where rabies is endemic in domestic dog populations [5]. However, the impacts of canine rabies are often overlooked, largely because human rabies deaths are now extremely rare in Western Europe and North America, where mass vaccination successfully eliminated the disease from domestic dog populations [6]. Increasing incidence of canine rabies in Africa and Asia has prompted concerns that similar strategies may not be effective in these areas [7,8]. The critical question now is whether global elimination of domestic dog rabies is achievable. Keys to answering this question include: a quantitative understanding of the transmission dynamics of rabies in domestic dog populations, particularly the basic reproductive number, R_0_; a quantitative understanding of domestic dog demography; and information about the practicality and effectiveness of various vaccination strategies. While recent data support the feasibility and practicality of domestic dog vaccination strategies [9–11], there are very little quantitative data on rabies transmission dynamics [12] and the underlying demographic processes.

Transmission is the most important process underlying infectious disease dynamics [13], but it is also the least understood. Rates of transmission are usually inferred from population patterns of disease incidence, but population-level analyses do not capture between-individual variation in transmission resulting from differences in behaviour, genetics, immune status, and environmental and stochastic factors, which play an important role in determining disease dynamics [14,15]. Contact tracing has been used to directly measure case-to-case transmission, and applications of the technique to emerging infections such as SARS have generated important insights into disease transmission and control in human populations [16,17], but transmission processes for diseases circulating in animal populations are much harder to study.

Rabies is an acute viral encephalitis that is spread through the saliva of infected hosts [2]. Clinical manifestations vary, but the neurological phase often includes increased aggression and the tendency to bite and thereby transmit infection; rapid progression to death is inevitable [4]. These distinctive signs make transmission of rabies easier to track than that of most other diseases and provide an unusual opportunity to explore epidemiological patterns at the scale of the individual.

**Figure 1:**
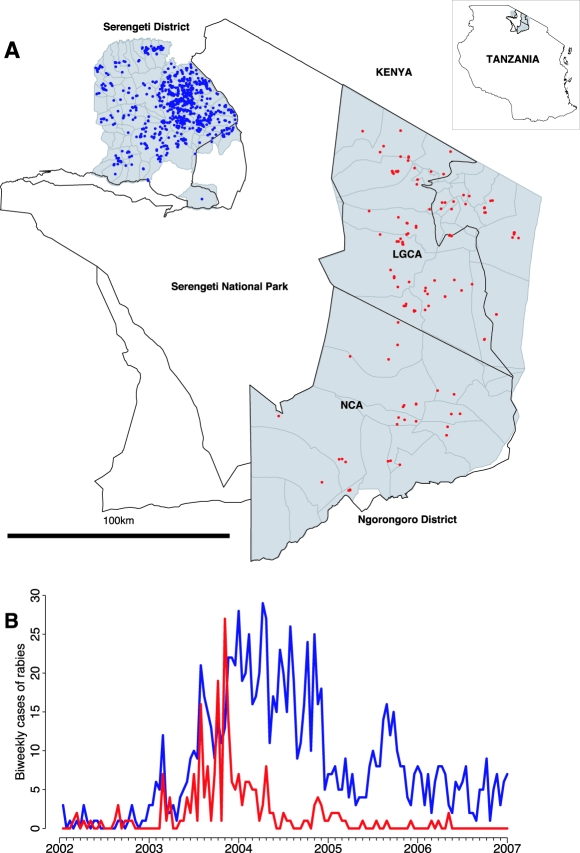
The Location and Timing of Animal Rabies Cases in Serengeti and Ngorongoro Districts, Northwest Tanzania (A) Rabies cases in Serengeti (blue) and Ngorongoro (red) districts from January 2002 until December 2006. LGCA = Loliondo Game Controlled Area, NCA = Ngorongoro Conservation Area. Dark gray lines show village boundaries. Populations of humans and domestic dogs are denser in Serengeti district than Ngorongoro (Table 3). (B) Biweekly time series of rabies cases in each district.

Here, we present data on rabies transmission in two districts of rural Tanzania, Serengeti and Ngorongoro (Figure 1). We were able to monitor the spread of infection using contact-tracing methods, which were feasible due to the discrete and memorable nature of transmission events. We recorded >3,000 potential transmission events between 2002 and 2006 and reconstructed case histories of over 1,000 suspect rabid animals that illustrate heterogeneity in several aspects of transmission, including the latency, movement patterns, and biting propensity of infected individuals. Although these districts border the Serengeti ecosystem, we have argued that domestic dogs are the sole maintenance population of rabies in this community: they make up over 90% of our observations of rabid animals, and the >70 isolates that have been sequenced (from 13 host species) are all consistent with the Africa 1b canid strain [18,19]. This is one of the most extensive datasets on individual transmission events assembled in an animal population; it has potential to shed light on critical, but often elusive, details of infectious disease transmission. We also analyze data from rabies outbreaks around the world, which provide a global and historical context for the Tanzania dataset.

## Results

### Epidemiological Parameters and Transmission

**Figure 2:**
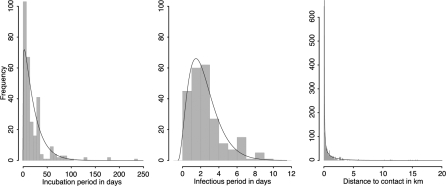
Observed Frequency Distributions of Important Epidemiological Parameters (A) The incubation period, (B) the infectious period, and (C) the spatial infection kernel. The best fitting gamma distributions to the data are shown by black lines (see Materials and Methods).

**Figure 3:**
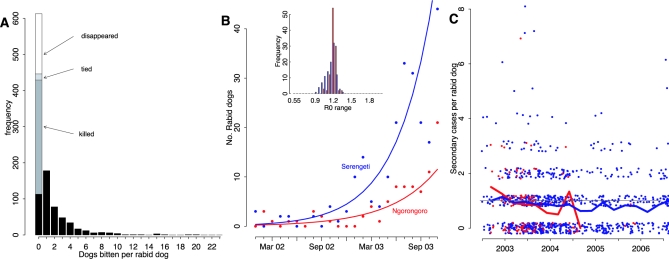
Transmission of Rabies (A) The distribution of dogs bitten per rabid dog (fitted by a negative binomial distribution with mean = 2.15 [95% CI: 1.95–2.37]; variance = 5.61 [95% CI: 4.63–6.92]; shape parameter *k* = 1.33 [95% CI: 1.23–1.42]; R_0_ ∼ 1.1). To calculate R_0_, we excluded dogs that were killed, tied, or those that disappeared before biting any other dogs. Variability in biting behaviour means that a small number of individuals disproportionately affect transmission and can potentially spark an epidemic, but since most individuals cause few, if any, infections, R_0_ is low and most introductions quickly die out (Figure 4C). (B) Exponential epidemic growth in Serengeti (blue, R_0_ ∼ 1.2) and Ngorongoro (red, R_0_ ∼ 1.1) districts. The R_0_ estimates from the epidemic trajectories were relatively insensitive to the period used for fitting the exponential curve. The inset shows the distribution of R_0_ estimates based on fitting to different regions of the time series. (C) The effective reproductive number, R, (averaged over three-month intervals) for Serengeti (blue) and Ngorongoro (red) districts measured from reconstructed epidemic trees that incorporate prior knowledge on who infected whom. Dots indicate the number of secondary cases resulting from each primary case (inferred from the composite tree of most likely links, with random jitter to avoid superposition on the *y*-axis). R_0_ estimated from these reconstructions (during the period of exponential epidemic growth) was ∼1.1 and ∼1.3 for Serengeti and Ngorongoro, respectively.

Analyses of the contact-tracing data generated robust estimates of epidemiological parameters that have important implications for rabies control (Table 1, Figures 2 and 3, and Figure S1) and provide insight into how infectious disease transmission scales from individual behaviour to population-level dynamics. We estimated R_0_ for rabies in Serengeti and Ngorongoro districts directly from infectious histories, from reconstructed epidemic trees based on the spatiotemporal proximity of cases, and from the exponential rate of increase in cases at the beginning of an epidemic. Biting behaviour of rabid dogs during the course of infectious periods was highly variable (mean bites per rabid dog = 2.15, 95% confidence interval (CI) from fitting a negative binomial distribution: 1.95–2.37; variance = 5.61, CI: 4.63–6.92; shape parameter *k* = 1.33; CI: 1.23–1.42) (Figure 3A). The probability that an unvaccinated dog developed rabies after being bitten by an infectious animal was high (*P*_rabies|bite_ = 0.49, CI: 0.45–0.52) (Table 1) if the bitten dog was not vaccinated or killed immediately after exposure. Multiplying the average number of dogs bitten per rabid dog by the probability of developing rabies following exposure gave an R_0_ estimate of 1.05 (CI: 0.96–1.14) (Figure 3A and Table 1). These estimates should be regarded as lower bounds, because not all transmission events were observed (this calculation excludes rabid dogs that were killed before biting other animals or that disappeared and likely corresponded to unknown or unobserved rabid dogs in other areas; see Materials and Methods). Detailed data on the timing and location of transmission events and infections allowed us to estimate the spatial infection kernel and generation interval (distances and times between source cases and their resulting infections, respectively) (Figure 2) and probabilistically reconstruct transmission networks (Videos S1 and S2). Calculating the average number of secondary cases per rabid dog during the period of exponential epidemic growth (before vaccinations were implemented) from these reconstructions gave similar R_0_ estimates of 1.1 in Serengeti district and 1.3 in Ngorongoro (CIs: 1.04–1.10 and 1.26–1.42, respectively) (Table 1). The more traditional approach of estimating R_0_, by fitting a curve to incidence data over the same interval of exponential epidemic growth, also produced similar estimates of 1.2 in Serengeti and 1.1 in Ngorongoro (CIs: 1.12–1.41 and 0.94–1.32, respectively) (Table 1 and Figure 3B). This approach is robust to underreporting (Text S1 and Figure S2) but should likewise be considered a lower bound, because some local control measures were instituted (such as tying or killing). We also estimated R_0_ from the intrinsic growth rate of outbreaks of domestic dog rabies elsewhere in the world (Table 2) and obtained values between 1.05 and 1.85, which are consistent with our estimates from northwest Tanzania.

**Table 1:**
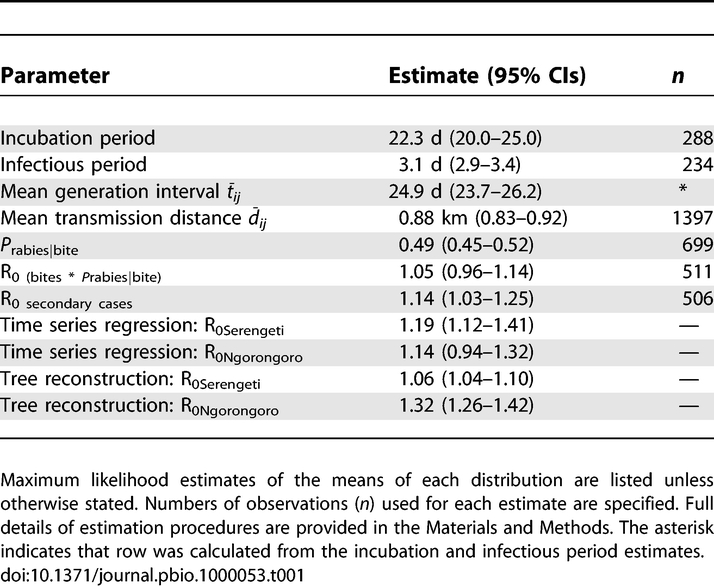
Epidemiological Parameter Estimates

**Table 2:**
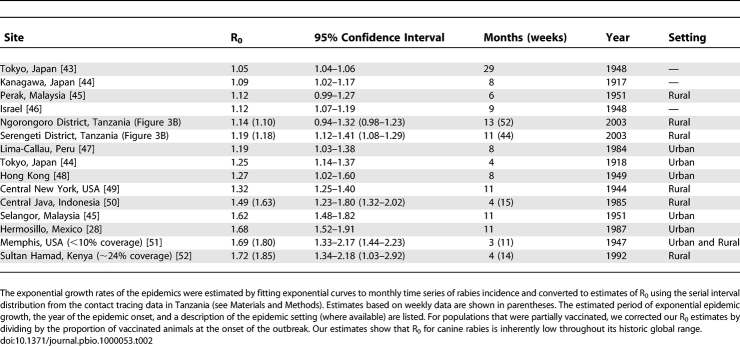
Estimates of R_0_ for Outbreaks of Rabies in Domestic Dog Populations around the World

**Table 3:**
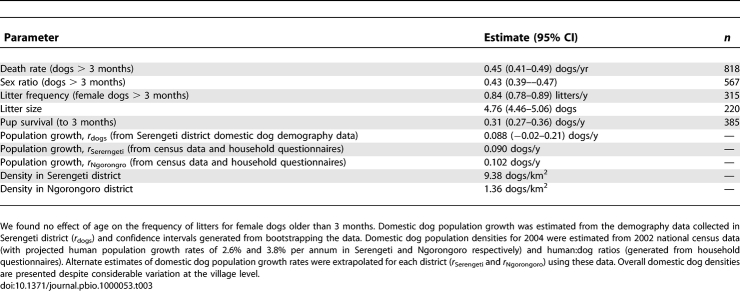
Demographic Parameters and Population Attributes Estimated from Domestic Dog Populations in Northwest Tanzania

**Figure 4:**
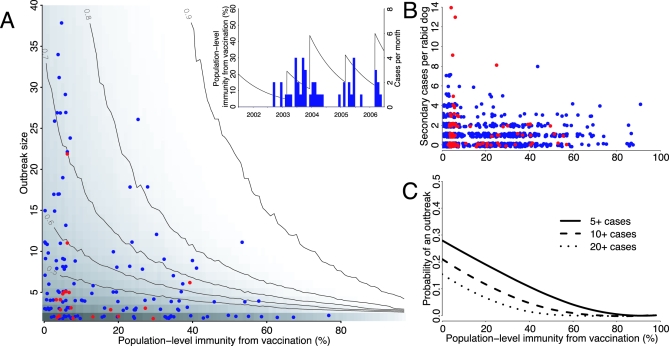
The Impact of Vaccination on Transmission (A) The size of village-level outbreaks (defined as at least two cases not separated by more than one month, isolated cases are assumed to be non-persistent introductions) in Serengeti (blue, *n* = 138) and Ngorongoro (red, *n* = 20) districts plotted against village-specific vaccination coverage at the outbreak onset. Coverage was extrapolated from a demographic model initialized with village-specific dog population estimates and incorporating village-specific vaccination data. Gray shading and contours correspond to the probability of observing an outbreak of a particular size or less, generated from 10,000 stochastic simulations of rabies transmission for every initial vaccination coverage (contours were calculated conditional upon >1 secondary case occurring). The inset illustrates a village-level example of the susceptible reconstruction used to calculate instantaneous vaccination coverage plotted beside rabies cases in that village. (B) The distribution of secondary cases per infectious dog as inferred from reconstructed epidemic trees in Serengeti (blue) and Ngorongoro (red) districts, plotted against vaccination coverage in the village where the primary case occurred. Random jitter was added to prevent superposition on the *y*-axis. (C) Probability of an outbreak being seeded by an introduced case under different levels of vaccination coverage. Due to heterogeneity in the transmission process outbreaks rarely occur when coverage is maintained above *P*_crit_. However if infections are frequently imported from outside the vaccinated region, at least 40% coverage would need to be maintained to reduce the probability of subsequent outbreaks (of at least ten cases) to <0.05.

For many diseases, R_0_ is expected to increase with host density [12,13,20,21]. Despite the domestic dog population density in Serengeti (9.38 dogs/km^2^) being considerably higher than the dog population density in Ngorongoro (1.36 dogs/ km^2^, see Table 3), we were unable to detect significant differences in our estimated values of R_0_ between the two districts. Nor did we find any conspicuous differences in R_0_ estimated from the outbreaks listed in Table 2, which represent a wide range of population densities. There may, in fact, be no relationship between R_0_ and population density for canine rabies. On the other hand, a subtle relationship between dog density and transmission rates might be difficult to detect for a number of reasons. To investigate whether it would be possible to decipher systematic differences in R_0_ across the range of values that we estimated, we simulated outbreaks using our epidemiological parameter estimates, but varied R_0_ (from R_0_ = 1 to R_0_ = 2), whilst maintaining individual variance in biting behaviour (same shape parameter *k*, see Text S2). Although the mean estimates of R_0_ from fitting to these simulated trajectories were accurate, they were surrounded by wide confidence intervals (Figures S2 and S5), suggesting that if only a small number of epidemics were sampled, any underlying relationship might not be apparent.

### Impacts of Interventions

Several mass domestic dog vaccination campaigns were carried out in villages in the study districts during the 5-y period. We analysed the impacts of these interventions at the village level to capture the wide variation in achieved levels of vaccination coverage. We incorporated demographic processes (Table 3 gives demographic parameter estimates) and waning of vaccine-induced immunity (see Materials and Methods), because these affect the level of herd immunity within the population at any one time. There were no rabies outbreaks (defined as at least two cases not interrupted by an interval of more than one month) in villages when vaccination coverage exceeded >70%. Small outbreaks occurred in villages with lower coverage and the largest (and longest) outbreaks only occurred in villages with <20% coverage. Observed outbreak sizes were within the range expected from the heterogeneity of biting behaviour and the coverage achieved by village-level vaccination campaigns (Figure 4A).

The effective reproduction number R, which describes transmission once an epidemic is underway, declined during the course of the observed epidemics (Figure 3C). At the level of individuals, vaccination coverage reduced the number of secondary cases per rabid dog (Figure 4B). More than 300 vaccinated dogs were identified by contact tracing as having been bitten by rabid animals. Only ten of these animals showed any signs indicative of rabies, although in the absence of vaccination approximately 50% (*P*_rabies|bite_ = 0.49) (Table 1) of these would have been expected to succumb to the disease. Individual actions by dog owners such as tying or killing exposed or infectious animals also had an impact. By killing rabid dogs, villagers reduced the overall average infectious period by around 16% (3.7 d for rabid animals that died from the disease versus 3.1 d for all infected animals, including those that were killed). However, there were no consistent declines through time in the number of bites by rabid dogs (Figure S3). Thus we consider vaccination to have been the overwhelming factor in curtailing the outbreaks (Figure 4A).

From our estimates of R_0_, we calculate the deterministic critical vaccination threshold for rabies elimination in rural Tanzania to be only 20% (*P*_crit_ = 1 – 1/R_0_), and even in areas where R_0_ is higher, *P*_crit_ rises to just 40% (Table 2). Our observations and simulations (Figure 4) demonstrate that small outbreaks occur by chance even when coverage exceeds *P*_crit_ and should be expected more frequently when there is individual variation in transmission (Text S2). Herd immunity declines rapidly in the interval between vaccination campaigns because of births and deaths in the domestic dog population (Figure 4, inset). To maintain herd immunity above *P*_crit_ between campaigns, therefore, requires a larger proportion of the dog population, *P*_target_, to be vaccinated (*P*_target_ = e^(ν*+d+r*)*T*^ *P*_crit_, where *r* is the rate of dog population growth, *d* is the death rate, 1/ν is the duration of vaccine-induced immunity, and *T* is the interval between campaigns (see Materials and Methods)). By incorporating demographic parameters (Table 3), we estimate that annual campaigns should therefore aim to vaccinate 60% of the dog population to avoid coverage falling below *P*_crit_.

## Discussion

The basic reproductive number, R_0_, is the average number of secondary infections produced by an infected individual in an otherwise fully susceptible population [20]. R_0_ is the most important parameter in infectious disease epidemiology, and considerable effort has been devoted to its estimation and to understanding its implications for disease control [20,22–26], although it is important to note that some factors not incorporated in R_0_, e.g., host births as well as deaths, may also have important control implications.

Depending upon the quality and quantity of data, a number of approaches can be used to estimate R_0_. Choosing the most appropriate method and assessing its accuracy can be difficult, given the associated assumptions and shortcomings [22]. Most methods do not account for variability in the pathogenesis and behaviour of infected animals; some methods make inferences from quantities that are confounded by (often unmeasured) responses to disease incidence (e.g., epidemic size or prevalence at equilibrium); and different methods are variously biased due to measurement and process error. Although our attempts to estimate R_0_ are also imperfect, they do incorporate individual variation in behaviour and pathogenesis, explicitly address several common assumptions, and have been carefully checked for biases through extensive simulations. The overall consistency in the low values of R_0_ that we estimated (∼1.1 < R_0_ < 2) is therefore reassuring and provides optimism for the feasibility of canine rabies control by vaccination.

If R_0_ increases with host density in this system, different threshold levels of vaccination coverage would be necessary to eliminate disease in different density populations [12,20]. However, our data on individual variation in biting behaviour also illustrate that it would be difficult to detect statistical differences in the range of R_0_ values that we estimated (Figure S2). Thus in practice, when only a small number of epidemics are observed, individual variation in transmission may mask any underlying variation in R_0_ driven by population density. So although we cannot decipher the relationship between population density and rabies transmission, the consistency of our individual- and population-level estimates from Tanzania and from a wide range of sites around the world allow us to estimate the threshold vaccination coverage necessary to eliminate the disease.

Our estimates of R_0_ predict that only relatively low levels of vaccination coverage are required to eliminate rabies (∼20–45%), but there is considerable variation in empirically observed levels of coverage that have successfully controlled the disease; low levels of coverage (30–50%) have been successful in some circumstances [27], although higher levels have also failed [28]. Our analyses suggest that these inconsistencies are, in large part, a consequence of host demography. When vaccinations are carried out in pulses, births and deaths within the host population will continuously reduce the level of herd immunity attained during campaigns (Figure 4, inset). Turnover of domestic dogs in rural Tanzania is very high (Table 3); therefore, annual campaigns should aim to vaccinate 60% of the dog population to maintain vaccination coverage above *P*_crit_ for the duration of the interval between campaigns. When successive campaigns have achieved this, rabies incidence has declined dramatically despite high endemic levels in adjacent areas [29]. Domestic dog population turnover therefore appears to have had a marked influence on rabies dynamics that explains the variable success of vaccination efforts. The empirically derived consensus that 70% coverage is sufficient for long-term rabies elimination [30,31] was likely reached because it is effective as a target for annual campaigns in almost all demographic settings, including those with particularly high turnover such as those we describe from Tanzania.

There are other potential explanations and caveats. The nutritional and health status of animals might affect the development of protective immunity in response to vaccination. However, more than 97% of dogs sampled from Serengeti district developed strong antibody titres (>0.5 IU/ml) in response to vaccination [32], suggesting that these factors do not impair the efficacy of dog vaccination in rural Tanzania. In addition, numerous practicalities—such as occasional failures in the cold chain, improper vaccination of animals, mistaken registrations, etc.—will all reduce the level of population immunity below the estimated vaccination coverage. Furthermore, our observations and simulations confirm that small outbreaks may occur simply by chance even when coverage exceeds *P*_crit_ [33], and these are particularly likely when there is individual variation in transmission (Figure 4). Higher levels of coverage are therefore necessary to reduce the chance of outbreaks with greater certainty; especially where the risk from imported infections is highest (Figure 4C). This could be a concern if canine rabies were to be eliminated from domestic dog populations but continued to circulate in sympatric wildlife; however, canine rabies was successfully eliminated in Western Europe and North America despite the presence of wildlife hosts capable of transmission.

Thousands of people die every year from this horrific and preventable disease, because the control of canine rabies has been severely neglected in developing countries [2]. Inherent inter-annual periodicity of epidemics exacerbates the situation, with rabies only intermittently perceived as problematic [6], as illustrated by the recent outbreak in China [34]. The problem of canine rabies has often been considered intractable in rural Africa, because of poor infrastructure, limited capacity, and the misperception that large populations of wild carnivores are responsible for disease persistence. Our analyses show that global control of canine rabies is entirely feasible and that successful elimination of canine rabies in many parts of the world has likely been achieved precisely because R_0_ is so low and institutional commitment to maintain high levels of vaccination coverage has been sustained [6]. Achieving vaccination coverage of 60% or more in dog populations in Africa is both logistically and economically feasible through annual vaccination campaigns [9–11,29]. The resultant reduction in costs of human post-exposure prophylaxis suggest that vaccination interventions targeted at domestic dog populations could translate into appreciable savings for the public health sector [3,8,29]. Furthermore, the inherently low R_0_ and the tractability of rabies contact-tracing indicates that once endemic rabies is controlled, elimination could be achieved through active case detection in remnant foci of infection (much like the strategy used to eradicate smallpox [35]); similar measures are proving effective in programmes to eliminate canine rabies in the Americas [36]. However, the most crucial step towards global elimination of canine rabies will be sustained commitment and coordinated efforts to maintain sufficient vaccination coverage in domestic dog populations.

## Materials and Methods

### Study areas.

We collected data from two districts in northwest Tanzania: Serengeti, inhabited by multi-ethnic, agro-pastoralist communities and high-density dog populations, and Ngorongoro, a multiple-use controlled wildlife area, inhabited by low-density pastoralist communities, predominantly Maasai, and lower-density dog populations (Figure 1). Attributes of the dog populations in these districts are presented in Table 3. Wildlife populations also differ in the two districts, but domestic dogs are the focus of this study because they are the only maintenance population of rabies in the area [18].

### Incidence data.

Data on patients with animal-bite injuries from hospitals and dispensaries, case reports of rabid animals from livestock offices, and community-based surveillance activities were used as primary sources [18]. Visits were made to investigate incidents reported in 2002 to 2006 involving suspected rabid animals. Cases were mapped at the site of the incident (wherever possible) and villagers interviewed to evaluate the status of the biting animal, determine its case history, and identify its source of exposure and subsequent contacts (if known). The same procedure was exhaustively followed for all associated exposures/cases. Interviews were conducted with veterinary officers, local community leaders, and livestock field officers in attendance, resulting in an active reporting network. Cases were diagnosed on epidemiological and clinical criteria, adapting the “six-step” method through retrospective interviews with witnesses [37]. Rabies was suspected if an animal displayed clinical signs [37] and either (a) disappeared or died within 10 days, or (b) was killed, but had a history of a bite by another animal or was of unknown origin. Additional clinical criteria for wild carnivores (∼10% of human exposures were caused by wild animals and ∼10% of inferred transmission events involved rabid wildlife) included tameness, loss of fear of humans, diurnal activity (for nocturnal species), and unprovoked biting of objects and animals without feeding. When multiple incidents involving suspected rabid wildlife were reported on the same/consecutive days within neighbouring homesteads, we assumed a single animal was involved.

Brain samples were collected and tested for confirmation wherever possible, but despite efforts to obtain diagnostic samples, most cases reported here were suspected rather than confirmed. Inadequate sample preservation such as storage at room temperature and long intervals between sample collection and testing (during which samples underwent repeated freeze-thaw cycles) probably caused specimens to deteriorate. Composite samples of each brain necessary to achieve the highest test reliability were also rarely available. Nevertheless, a high percentage of samples from suspected cases of rabies were confirmed by laboratory diagnosis (∼75%) suggesting that use of epidemiological and clinical criteria is justified and reliable [18]. Researchers are encouraged to contact the authors regarding data availability.

### Vaccination data.

Dog vaccination campaigns in Serengeti district in 2000 resulted in low and patchy vaccination coverage (35–40% estimated from post-vaccination household surveys). Annual campaigns conducted from 2003 onwards in a 10-km zone adjacent to the western border of Serengeti National Park achieved higher coverage levels of between 40 and 80%. In 2004, the Tanzanian government conducted vaccinations in villages in Serengeti district beyond the 10-km zone reaching 55% coverage across the remainder of the district, but in subsequent years, campaigns were less systematic and conducted in fewer villages. Vaccination in Ngorongoro was restricted to small-scale localised campaigns in the district town centre until 2004, whereupon widespread annual vaccinations were implemented with overall coverage exceeding 80% [9]. Data on the number of dogs vaccinated in each village and on each campaign date were collected from 2003 onwards.

### Parameter estimation.

The incubation period and duration of infectiousness were estimated for rabies in domestic dogs from records of when individual dogs were bitten, developed clinical signs, and were killed or died. Gamma distributions were fitted to these data using maximum likelihood with interval censoring to account for cases where the relevant data were only approximately known (Figure 2 and Table 1). To estimate the probability distribution of the generation interval, *G*(*t*), an incubation and an infectious period were drawn from their respective distributions, a “time-to-bite” deviate was drawn from a uniform distribution over the interval of the infectious period, and the two intervals were summed. There was a significant correlation between the length of the infectious and incubation periods, but significance was entirely due to a single data point; we therefore treated the distributions as independent. The spatial infection kernel *K*(*d*) was estimated by fitting a gamma distribution to the distances between known source cases and animals that they contacted. Many contacts occurred within the same, or neighbouring, homesteads. In these cases, the precise distance was not always recorded, but we assumed it was less than 100 m. We therefore replaced the probability of a contact within 100 m by the probability distribution averaged over the range 0–100 m.

### The basic reproductive number R_0_.

(*1*) *Direct estimates from infectious histories.* Using maximum likelihood, we fitted a negative binomial distribution to data on biting behaviour of rabid dogs (Figure 3A). The probability of developing rabies following a bite (*P*_rabies|bite_) was estimated, excluding bitten animals that had previously been vaccinated, or that were either killed or vaccinated immediately after the bite, and binomial confidence intervals were calculated. R_0_ was estimated as the probability *P*_rabies|bite_ multiplied by the average number of bites per rabid dog and confidence intervals were calculated using a resampling procedure. Dogs that were removed (killed or tied up) before causing secondary cases in other dogs (even if they bit people) were excluded from this calculation, as were suspect rabid dogs that either disappeared before biting other dogs or that were of unknown origin and were killed before being observed to bite other dogs (Figure 3A). We pooled data from both districts for this estimate because insufficient complete case-histories of rabid dogs (after excluding cases with interventions) were traced to accurately estimate R_0_ for Ngorongoro (35 versus 477 in Serengeti). We also estimated R_0_ directly from the distribution of secondary cases per rabid dog. Dogs that were bitten by rabid animals but did not develop rabies because of interventions (previous vaccination or being killed/vaccinated immediately after the bite) were multiplied by *P*_rabies|bite_ and added to observed secondary cases, giving an expected number of secondary cases per rabid dog in the absence of intervention and a similar estimate of R_0_ (1.14, CI: 1.03–1.25) (Figure S1).

(2) *Epidemic tree reconstruction.* We used an algorithm for probabilistically constructing epidemic trees based on the location of cases in space and time [38]. For each suspected case (*i*), we chose a progenitor (*j*) at random with probability *p_ij_* from all *n* cases preceding that case, where:

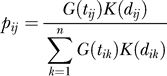

*G* is the distribution of generation times, *t_ij_* is the length of time (in days) between the occurrence of case *i* and its potential progenitor *j* (*G*(*t*) = 0 for *t* < 0), *K* is the spatial infection kernel, and *d_ij_* is the distance (in km) between the locations of case *i* and its potential progenitor *j* (using the average probability when distance <100 m, see above). Because the dates that some individuals were bitten or developed rabies were only approximately known, 1,000 bootstrapped datasets were generated with the dates drawn randomly from a uniform distribution over the window of uncertainty and a consensus tree of the most probable links was determined and used to generate secondary case distributions illustrated in Figure S1. Because transmission of rabies from livestock is recorded extremely rarely, we did not allow livestock progenitors, which considerably improved the match between known and assigned links compared to an algorithm where all species could be assigned as progenitors. All detected cases in carnivores (including domestic cat and wildlife cases) were included in the tree reconstructions using the spatial infection kernel and generation interval parameters estimated for domestic dogs. The contribution of nondomestic dog carnivores to the overall epidemic was small, and estimates of within- and between-species transmission are described elsewhere [18]. When known links between primary and secondary cases were not retained in the trees, they were correctly assigned in more than 60% of cases in both districts, indicating that probabilistic reconstruction was effective. The average number of secondary cases putatively produced from each primary case was calculated from the bootstrapped trees. R_0_ was estimated as the average number of infections caused per rabid dog that was infectious during the period of exponential epidemic growth. Determining the period of exponential growth is somewhat subjective; for consistency between methods, we used the interval that gave the median R_0_ value for time series regression estimates (see below). The choice of interval caused more variance in R_0_ estimates for this reconstruction technique than for other methods because it averages the heterogeneous behaviour of a small number of individual animals that spark an epidemic. Thus inclusion or exclusion of particularly infectious individuals has a large effect on R_0_.

(3) *Inference from the epidemic curve.* A single infection will cause future cases distributed according to the probability distribution of the generation interval. Therefore the number of cases arising in any given interval is the result of those cases that occurred at times in the past whose secondary cases occur in this interval and is determined by the probability distribution of the generation interval. This intuitive description is formalized by the Euler-Lotka equation, adapted for an infection process [25] and an expression for R_0_ can be obtained:

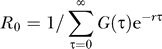
We estimated the initial growth rate of the epidemic (*r*) by fitting an exponential curve to incidence data using a generalized linear model. We compared Akaike's Information Criterion values to determine the appropriate error structure (Poisson or negative binomial). The choice of which part of the epidemic curve the model should be fit to was subjective, therefore the model was fit to all possible sections of the epidemic curve (using a minimum of nine consecutive months) and the median, the 2.5th and the 97.5th percentile of the R_0_ estimates are presented in Table 1. Figure 3B (inset) shows that the estimate of R_0_ was robust to the interval chosen for fitting the curve. We used the same method to estimate R_0_ from data that we had compiled on outbreaks of canine rabies from elsewhere in the world. For these time series, we fitted exponential curves to the intervals between the first recorded case and the month (or week) with highest rabies incidence (Table 2) and converted the estimated growth rates to estimates of R_0_ using the serial interval distribution data gathered by contact tracing in Tanzania. For partly vaccinated populations, we corrected our R_0_ estimates by dividing by the fraction of dogs which were vaccinated prior to the outbreak [12]. For all the outbreaks considered, including those in Tanzania, some localized and individual control measures may have been instituted (such as tying up or killing infected animals), and therefore our R_0_ estimates should be regarded as lower bounds. However simulations also revealed that for very low values of R_0_ (<1.2), estimates from the epidemic trajectory can be slightly biased upwards (Figure S2). This is probably because at very low levels of R_0_, most introductions do not initiate further cases and therefore a small number of individuals with higher than average biting behaviour are needed to trigger epidemics, thus biasing trajectories.

### The effective reproductive number R.

The effective reproductive number R measures the average number of secondary cases per primary infection once an epidemic is underway. R changes through space and time depending upon the implementation of control measures, the depletion of susceptibles and the build-up of local correlations in the spatial distribution of infected and susceptible individuals. Numbers of secondary cases per rabid dog (inferred from the epidemic tree reconstructions) were calculated monthly and averaged across bootstrapped trees to give a time-varying estimate of R (Figure 3C). Although R declined through time in both districts, there was no apparent temporal trend in the biting behaviour of rabid dogs (Figure S3), suggesting that domestic dog vaccination was the main factor responsible for reducing transmission.

### Domestic dog demography.

To calculate vaccination coverage and the decline in herd immunity due to population turnover and waning of vaccine-induced immunity, it was necessary to estimate the size of the domestic dog populations (*N*) and their rates of growth (*r*_dogs_). We projected human population sizes in both districts using 2002 national census data [39,40], and we calculated human:dog ratios from household questionnaires conducted in 1994, 2003, and 2008 in Serengeti district and in 1994 and 2004 in Ngorongoro district. We then estimated dog populations from the projected human population sizes and the human:dog ratios and calculated the rate of increase of the dog population in each district (*r*_dog*s*_ = log(*N_t_*/*N_0_*)/*t*) (Table 3).

An alternative estimate of the rate of domestic dog population growth was derived from demographic data collected using household questionnaires. The death rate of dogs (*d*) was calculated using a Cox proportional hazards model of survival from longitudinal data (*n* = 802). When pups (dogs under 3 months of age) were excluded from the model, neither age nor sex significantly affected survival. The per-capita birth rate (*b*) was assumed to be the product of the sex ratio (ρ), the average litter size (*l*), and frequency (φ) and pup survival (*s*) (*b* = ρ*l*φ*s*). These demographic parameters were estimated from cross-sectional data (309 litters) and the rate of increase was calculated (*r*_dogs_ = *b – d*). Pup survival was estimated from a subset of puppies that remained in the household, because of the unknown fate of puppies that were given away or sold. We suspect that mortality of female puppies is greater than males. However, obtaining reliable data to accurately estimate pup survival is difficult, and the result of assuming equal mortality rates is an estimate of *r*_dogs_ that is more conservative with respect to vaccination coverage (i.e., results in lower population-level immunity). This estimate of *r*_dogs_ (0.088 dogs/y) was similar to other estimates from the region [41,42] and close to those calculated directly from population sizes (*r*_Serengeti_ = 0.090 dogs/y, *r*_Ngorongoro_ = 0.102 dogs/y) (Table 3). A comparison of the stable age distribution (calculated from cross-sectional data assuming a roughly constant rate of population growth) was consistent with age distributions predicted from the estimated demographic parameters.

### Analysis of the impacts of intervention.

To evaluate whether the predicted level of vaccination coverage required to control rabies (*P*_crit_ = 1 – 1/R_0_) was sufficient in practice [20], we plotted the size of village-level outbreaks (an outbreak was defined as at least two cases not interrupted by an interval of more than one month) against vaccination coverage in that village at the time of the case that initiated the outbreak.

Vaccination coverage was modeled by susceptible reconstruction using demographic parameters described above (we show the results from using the largest estimate of *r*_dogs_ (0.10 dogs/y) because this gives the most conservative predictions of the impacts of vaccination, but results are very similar using the lower *r*_dogs_ estimates). We assumed coverage was approximately 20% in January 2002 and that the duration of vaccine-induced immunity (1/ν) was approximately 3 y (http://www.intervet.co.uk/Products_Public/Nobivac_Rabies/090_Product_Datasheet.asp). Numbers of vaccinated and susceptible animals within a village were adjusted according to the doses of vaccine used at village vaccination stations on each campaign date (sufficient vaccine was provided such that all animals brought to the station could be vaccinated). A time series of cases in a village and the associated susceptible reconstruction are shown in the inset of Figure 4A.

To predict the expected size of outbreaks given the observed variability in transmission, we simulated outbreaks in a starting population of 500 dogs (similar to the domestic dog population size in an average village); this choice had little effect on our results. We used our parameter estimates (Table 1) to randomly assign secondary cases and corresponding generation intervals. Each realization was seeded by a single animal and the starting population was initialized with vaccination coverage generated from a binomial distribution. For comparison with the outbreak data we conditioned each realization upon >1 secondary case (Figure 4A). Demographic parameters were incorporated, and 10,000 runs were completed for each starting condition. We also calculated the probability of an outbreak of a particular size or larger being seeded by one infectious case to evaluate the coverage needed to prevent outbreaks with different degrees of certainty (Figure 4C and Figure S4).

If *V* and *N* denote numbers of vaccinated individuals and the total population size respectively, then vaccination coverage can be expressed as a proportion *P* = *V*/*N*. The number of vaccinated dogs declines following a campaign as individuals die and as vaccine-induced immunity wanes (*V*_*t*_ = *V_0_*e*^–^* ^(*d+*ν)*t*^, where *d* is the death rate and 1/*ν* is the duration of vaccine-induced immunity), whereas the total population grows at the rate of population increase (*N*_*t*_ = *N*_0_e*^rt^*). To prevent sustained endemic transmission, vaccination coverage must be maintained above *P*_crit_ (such that R is held below 1). From our estimates of demographic parameters and R_0_, we calculated the proportion of the population that needs to be vaccinated, *P*_target_, to prevent vaccination coverage falling below *P*_crit_ during the interval, *T*, between campaigns: *P*_target_ = e^(ν*+d+r*)*T*^ *P*_crit_. This formulation for estimating the coverage needed to interrupt endemic transmission given turnover in the domestic dog population assumes that immunity from vaccination lasts an average of 1/ν time units and declines exponentially. In reality, vaccine-induced immunity is likely to be closer to a fixed duration, and thus fewer dogs would be expected to lose immunity during the 1-y interval between campaigns than under the exponential model. This indicates that our estimate of *P*_target_ may be slightly overestimated, although this is an important area for further investigation.

## Supporting Information

Figure S1 R_0_ Estimated from Secondary Case Distributions Observed numbers of secondary cases are shown in gray. We extrapolated additional cases (using the probability of developing rabies following a bite, 0.49) that would have occurred had there been no intervention (black). The inset shows the estimated secondary case distributions (∼1.1-1.3) for dogs in Serengeti (black) and Ngorongoro (red) districts based on the reconstructed trees during the early stages of the epidemics.
(3.92 MB EPS)

Figure S2 Accuracy of R_0_ Estimates Derived from Epidemic Trajectories (A) Estimates of R_0_ from fitting to trajectories of simulated epidemics plotted against the underlying R_0_ used in the simulations (biting behaviour was modelled using a negative binomial distribution, varying the mean number of bites per dog whilst keeping the shape parameter constant). The median R_0_ estimate from 1,000 realizations is shown by the solid black line and 95 percentiles are indicated by gray shading. R_0_ was estimated accurately across a range of underlying R_0_ values apart from at very low values (R_0_ < 1.2) when estimates were slightly inflated.
(B-E) Simulated time series were randomly resampled to test whether incomplete reporting biased the accuracy of R_0_ estimates. The value of R_0_ used to simulate epidemics is shown in the top right corner of each panel (B–E) and indicated by the dotted red line. The median estimated value of R_0_ (from 1,000 simulated, sampled epidemics) is indicated by the solid black line, and the interquartile range and 95 percentiles of R_0_ estimates are shaded in dark and light gray, respectively. Simulations illustrate that R_0_ estimates derived from fitting curves to outbreak time series are robust to underreporting and stable across a reasonable range of underlying R_0_ values although slightly overestimated at very low values (A and B).
(5.81 MB EPS)

Figure S3 Temporal Trends in Biting Behaviour and Secondary Cases Caused by Rabid Dogs Numbers of secondary cases (open circles: inferred from reconstructed epidemic trees) and bites (solid circles: estimated from contact tracing) per rabid dog averaged over three-month intervals are plotted for (A) Serengeti and (B) Ngorongoro. Secondary cases decreased in both districts, but there was no trend in the number of bites per rabid dog in Ngorongoro and a slight increase in Serengeti.
(5.86 MB EPS)

Figure S4 Impacts of Biting Heterogeneity on the Probability of Seeding an Outbreak The simulated proportion of outbreaks of a certain size (5, 10, or 20 cases) or greater that were seeded by an introduced case given biting behaviour described by a negative binomial with mean and variance equal to observed biting behaviour (solid lines) or a poisson with the same mean (dashed lines).
(5.57 MB EPS)

Figure S5 The Influence of Biting Heterogeneity on Epidemic Trajectories and Estimates of R_0_
The distributions of R_0_ estimates from fitting curves to simulated epidemic trajectories generated from biting behaviour described by a negative binomial distribution (black) with mean and variance equal to observed biting behaviour or by a poisson distribution (red) with the same mean. The range of R_0_ estimates from simulations spans the range of estimates from compiled outbreak data from around the world (Table 2). Considerably more variance in estimates was generated from negative binomial biting behaviour than from Poisson biting (>25% from monthly time series and >800% from weekly time series, upper 95% prediction intervals of 1.71 and 2.65, respectively, versus 1.65 and 1.69, respectively).
(5.58 MB EPS)

Text S1 Impacts of Under-Reporting and Incomplete Tracing on Estimation of R_0_
(24 KB DOC)

Text S2 Effects of Heterogeneity in Transmission Behaviour (22 KB DOC)

Video S1 Rabies Transmission in Serengeti District Inferred from Detailed Spatiotemporal Incidence Data and Estimated Epidemiological Parameters Rabies cases appear as red dots. Incubating animals appear as black dots, which turn red when clinical signs start (only animals that went on to develop rabies are shown). When a rabid animal bites another animal that will subsequently develop rabies, a black line connects the two individuals. The video is on a weekly timescale and the red arrow on the time series of rabies incidence corresponds to infectious cases during that week.
(2.20 MB AVI)

Video S2 Rabies Transmission in Ngorongoro District Inferred from Detailed Spatiotemporal Incidence Data and Estimated Epidemiological Parameters Rabies cases appear as red dots. Incubating animals appear as black dots, which turn red when clinical signs start (only animals that went on to develop rabies are shown). When a rabid animal bites another animal that will subsequently develop rabies a black line connects the two individuals. The video is on a weekly timescale and the red arrow on the time series of rabies incidence corresponds to infectious cases during that week.
(409 KB AVI)
