# Supplementary material for: Transmission Dynamics and Prospects for the Elimination of Canine Rabies
Source: PLoS Biol. 2009 Mar 10;7(3):e1000053. doi: 10.1371/journal.pbio.1000053 (PMC2653555; doi:10.1371/journal.pbio.1000053)
Supplement: Supplementary file 1 [file pbio.1000053.sd001.doc]

**Text S1**

*Impacts of under-reporting and incomplete tracing on estimation of R_0_*

Although surveillance effort was consistently high during the study, inevitably some cases were not reported or could not be identified and traced completely, which may have biased our estimates of R_0_. Data inspection indicates that few genuine human cases of rabies were missed; approximately 70% of patients from Serengeti and Ngorongoro districts who reported to medical facilities with bite injuries from animals suspected to be rabid or whose status was unclear were successfully traced. Most of the patients that we were unable to trace originated from the district town centres, and we suspect that most of these incidents involved healthy animals (as with most of the traced cases from these centres) and that patients reported to medical facilities largely because of their greater accessibility. Determining the number of rabid animals that were not observed is difficult. The average distance between directly linked cases (<1km) implies that few animals would have been entirely lost from the study districts (which are largely bounded by Serengeti National Park, the Mara River, Lake Natron and the Rift valley). The number of rabid dogs that disappeared (392) was close to the number of rabid dogs of unknown origin (385) and it is likely that many of these were the same individuals. We cannot exclude the possibility of missing intermediate cases, but the close correspondence between observed transmission pathways and inferred reconstructions suggests that surveillance quality was high.

We also tested whether underreporting affected the accuracy of R_0_ estimates calculated from epidemic trajectories by fitting to sampled simulated time series of rabies incidence (Figure S3). We found the procedure to be robust to underreporting (even when sampling only 20% of simulated incidence data, the underlying R_0_ value was accurately recovered; Figure S3B-E). In conclusion, the consistency between our R_0_ estimates from two alternative methods (measurements of rabid animal behaviour and inference from the incidence time series) strongly suggests that observation biases are small.
