# Supplementary material for: Transmission Dynamics and Prospects for the Elimination of Canine Rabies
Source: PLoS Biol. 2009 Mar 10;7(3):e1000053. doi: 10.1371/journal.pbio.1000053 (PMC2653555; doi:10.1371/journal.pbio.1000053)
Supplement: Supplementary file 2 [file pbio.1000053.sd002.doc]

**Text S2**

*Effects of heterogeneity in transmission behaviour*

We explored the phenomenological effects of individual-level variation on estimates of R_0_ by simulating epidemics from our estimated parameter distributions for biting behaviour and the probability of developing rabies following exposure. Specifically we examined the differences resulting from biting behaviour described by a negative binomial distribution versus a poisson distribution with the same mean.

Simulated epidemics had substantial variation in monthly and weekly case counts (much like the data). The probability that an introduced case initiated an epidemic was lower in simulations with realistic biting behaviour (negative binomially distributed) than with biting behaviour that was less variable (poisson distributed) [Figure S5]. In both cases the average R_0_ estimated from simulated epidemics (conditioned upon at least ten cases occurring) was equal to the individual-level R_0_ used to generate the epidemic (1.2); though 95% prediction intervals (PIs - from multiple realizations) varied and were greater for simulations with realistic biting behaviour (25% more variance in R_0_ estimates from negative binomially distributed biting behaviour versus Poisson, upper 95% PIs of 1.71 and 1.65 respectively). This variation was more pronounced if time series resulting from realizations were aggregated weekly, rather than monthly (more than eight times more variance in R_0_ estimates from simulations with negative binomial biting behaviour rather than Poisson, upper 95% PIs of 2.61 and 1.69 respectively) [Figure S6].

These results can be explained intuitively; variability in biting behaviour means that a small number of individuals disproportionately affect transmission and can potentially spark an epidemic, but since most individuals cause few, if any, infections (Figure 2A), R_0_ is low and most introductions quickly die out (Figure 3C). The same stochasticity in the transmission process means that it will be difficult to detect statistical differences in R_0_ that might be expected in populations of different densities because of the range of epidemic trajectories that can be expected by chance alone.
